# Supplementary material for: Species-Specific Analysis of Bacterial Vaginosis-Associated Bacteria
Source: Microbiol Spectr. 2023 Jun 22;11(4):e04676-22. doi: 10.1128/spectrum.04676-22 (PMC10434177; doi:10.1128/spectrum.04676-22)
Supplement: Supplemental file 1 — Supplemental material. Download spectrum.04676-22-s0001.pdf, PDF file, 0.7 MB [file spectrum.04676-22-s0001.pdf]

Job Title	BVAB-1_AY724739.1 Uncultured bacterium clone...
RID	PNJ0X2UG016 *Search expires on 11-10 04:20 am* Download All
Program	BLASTN Citation
Database	nt See details
Query ID	lcl|Query_40607
Description	BVAB-1_AY724739.1 Uncultured bacterium clone 123-f 57 ...
Molecule type	dna
Query Length	1007
Other reports	Distance tree of results MSA viewer

Filter Results

Organism *only top 20 will appear* exclude

Type common name, binomial, taxid or group name

+ Add organism

Percent Identity to **E value** to **Query Coverage** to

Filter Reset

Descriptions | Graphic Summary | Alignments | Taxonomy

Sequences producing significant alignments Download Select columns Show 100

select all *100 sequences selected* GenBank Graphics Distance tree of results MSA Viewer

Description	Scientific Name	Max Score	Total Score	Query Cover	E value	Per. Ident	Acc. Len	Accession
Clostridiales genomosp. BVAB1 isolate UAB071 chromosome, complete genome	Clostridiales genomosp. BVAB1	1860	7431	100%	0.0	100.00%	1649642	CP049781.1
Uncultured bacterium clone ncd1853a08c1 16S ribosomal RNA gene, partial sequence	uncultured bacterium	1860	1860	100%	0.0	100.00%	1352	JF160899.1
Uncultured bacterium clone nbu405d06c1 16S ribosomal RNA gene, partial sequence	uncultured bacterium	1860	1860	100%	0.0	100.00%	1352	GQ037632.1
Uncultured bacterium clone 123-f 57 16S ribosomal RNA gene, partial sequence	uncultured bacterium	1860	1860	100%	0.0	100.00%	1007	AY724739.1
Uncultured bacterium clone rRNA296 16S ribosomal RNA gene, partial sequence	uncultured bacterium	1860	1860	100%	0.0	100.00%	1507	AY959069.1
Uncultured bacterium clone nck189a08c1 16S ribosomal RNA gene, partial sequence	uncultured bacterium	1855	1855	100%	0.0	99.90%	1352	KF094234.1
Uncultured bacterium clone 23.001-2 16S ribosomal RNA gene, partial sequence	uncultured bacterium	1855	1855	100%	0.0	99.90%	1377	JX871285.1
Uncultured bacterium clone 22.001-2 16S ribosomal RNA gene, partial sequence	uncultured bacterium	1855	1855	100%	0.0	99.90%	1377	JX871284.1
Uncultured bacterium clone ncd1854c08c1 16S ribosomal RNA gene, partial sequence	uncultured bacterium	1855	1855	100%	0.0	99.90%	1352	JF159358.1
Uncultured bacterium clone nbw1010h11c1 16S ribosomal RNA gene, partial sequence	uncultured bacterium	1855	1855	100%	0.0	99.90%	1352	GQ047285.1
Uncultured bacterium clone nbu405e02c1 16S ribosomal RNA gene, partial sequence	uncultured bacterium	1855	1855	100%	0.0	99.90%	1352	GQ037635.1

Supplemental file 1.1: BLAST alignment analysis of BVAB-1

Download GenBank Graphics Sort by: E value

Clostridiales genomosp. BVAB1 isolate UAB071 chromosome, complete genome

Sequence ID: CP049781.1 **Length:** 1649642 **Number of Matches:** 4

Range 1: 9272 to 10278 GenBank Graphics ▼ Next Match ▲ Previous Match

Score	Expect	Identities	Gaps	Strand
1860 bits(1007)	0.0	1007/1007(100%)	0/1007(0%)	Plus/Plus

```
Query  1     TGGGGAATATTGCACAATGGGCGAAAGCCTGATGCAGCGACGCCGCGTGAGCGAAGAAGT  60
             ||||||||||||||||||||||||||||||||||||||||||||||||||||||||||||
Sbjct  9272  TGGGGAATATTGCACAATGGGCGAAAGCCTGATGCAGCGACGCCGCGTGAGCGAAGAAGT  9331

Query  61    ATTTCGGTATGTAAAGCTCTATCAGAAGGGAAGAAAATGACGGTACCTTACTAAGAAGCT  120
             ||||||||||||||||||||||||||||||||||||||||||||||||||||||||||||
Sbjct  9332  ATTTCGGTATGTAAAGCTCTATCAGAAGGGAAGAAAATGACGGTACCTTACTAAGAAGCT  9391

Query  121   CCGGCTAAATACGTGCCAGCAGCCGCGGTAATACGTATGGAGCAAGCGTTATCCGGATTT  180
             ||||||||||||||||||||||||||||||||||||||||||||||||||||||||||||
Sbjct  9392  CCGGCTAAATACGTGCCAGCAGCCGCGGTAATACGTATGGAGCAAGCGTTATCCGGATTT  9451

Query  181   ACTGGGTGTAAAGGGAGTGTAGGCGGCACTATAAGTCTGATGTGAAAACCTAAGGCTTAA  240
             ||||||||||||||||||||||||||||||||||||||||||||||||||||||||||||
Sbjct  9452  ACTGGGTGTAAAGGGAGTGTAGGCGGCACTATAAGTCTGATGTGAAAACCTAAGGCTTAA  9511

Query  241   CCATAGGATTGCATTGGAAACTGTAGAGCTGGAGTATCGGAGAGGCAAGCGGAATTCCTG  300
             ||||||||||||||||||||||||||||||||||||||||||||||||||||||||||||
Sbjct  9512  CCATAGGATTGCATTGGAAACTGTAGAGCTGGAGTATCGGAGAGGCAAGCGGAATTCCTG  9571

Query  301   GTGTAGTGGTGAAATACGTAGATATCAGGAAGAACATCGGTGGCGAAGGCGGCTTGCTGG  360
             ||||||||||||||||||||||||||||||||||||||||||||||||||||||||||||
Sbjct  9572  GTGTAGTGGTGAAATACGTAGATATCAGGAAGAACATCGGTGGCGAAGGCGGCTTGCTGG  9631

Query  361   ACGATAACTGACGCTAAGGCTCGAAAGCGTGGGAAGCGAACAGGATTAGATACCCTGGTA  420
             ||||||||||||||||||||||||||||||||||||||||||||||||||||||||||||
Sbjct  9632  ACGATAACTGACGCTAAGGCTCGAAAGCGTGGGAAGCGAACAGGATTAGATACCCTGGTA  9691

Query  421   GTCCACGCTGTAAACGATGAACACTAGGTGTTGGGAGGCTAAGCCTTTCAGTGCCGCAGC  480
             ||||||||||||||||||||||||||||||||||||||||||||||||||||||||||||
Sbjct  9692  GTCCACGCTGTAAACGATGAACACTAGGTGTTGGGAGGCTAAGCCTTTCAGTGCCGCAGC  9751

Query  481   AAACGCAATAAGTGTTCCACCTGGGGAGTACGTTCGCAAGAATGAAACTCAAAGGAATTG  540
             ||||||||||||||||||||||||||||||||||||||||||||||||||||||||||||
Sbjct  9752  AAACGCAATAAGTGTTCCACCTGGGGAGTACGTTCGCAAGAATGAAACTCAAAGGAATTG  9811

Query  541   ACGGGGACCCGCACAAGCGGTGGAGCATGTGGTTTAATTCGAAGCAACGCGAAGAACCTT  600
             ||||||||||||||||||||||||||||||||||||||||||||||||||||||||||||
```

Supplemental file 1.2: BLAST alignment analysis of BVAB-1

Description	BVAB-2_AY724740.1 Uncultured bacterium clone 123-f 23 ...
Molecule type	dna
Query Length	1009
Other reports	Distance tree of results MSA viewer

Percent Identity ___ to ___ E value ___ to ___ Query Coverage ___ to ___

Filter Reset

Descriptions | Graphic Summary | Alignments | Taxonomy

Sequences producing significant alignments Download Select columns Show 100

select all *100 sequences selected* GenBank Graphics Distance tree of results MSA Viewer

Description	Scientific Name	Max Score	Total Score	Query Cover	E value	Per. Ident	Acc. Len	Accession
Uncultured bacterium clone ncd989g05c1 16S ribosomal RNA gene, partial sequence	uncultured bacte...	1864	1864	100%	0.0	100.00%	1358	HM334697.1
Uncultured bacterium clone ncd997d08c1 16S ribosomal RNA gene, partial sequence	uncultured bacte...	1864	1864	100%	0.0	100.00%	1358	HM332642.1
Uncultured bacterium clone 123-f 23 16S ribosomal RNA gene, partial sequence	uncultured bacte...	1864	1864	100%	0.0	100.00%	1009	AY724740.1
Uncultured bacterium clone rRNA115 16S ribosomal RNA gene, partial sequence	uncultured bacte...	1864	1864	100%	0.0	100.00%	1513	AY958888.1
Oscillospiraceae bacterium strain CHIC02 1186E3-8 16S ribosomal RNA gene, partial sequence	Oscillospiraceae ...	1858	1858	100%	0.0	99.90%	1450	OP540273.1
Uncultured bacterium clone ncd1058g03c1 16S ribosomal RNA gene, partial sequence	uncultured bacte...	1858	1858	100%	0.0	99.90%	1358	HM344558.1
Uncultured bacterium clone ncd1008g01c1 16S ribosomal RNA gene, partial sequence	uncultured bacte...	1858	1858	100%	0.0	99.90%	1358	HM341223.1
Uncultured bacterium clone ncd988h05c1 16S ribosomal RNA gene, partial sequence	uncultured bacte...	1858	1858	100%	0.0	99.90%	1358	HM334584.1
Uncultured bacterium clone rRNA164 16S ribosomal RNA gene, partial sequence	uncultured bacte...	1858	1858	100%	0.0	99.90%	1514	AY958937.1
Oscillospiraceae bacterium strain UPII 610-J 16S ribosomal RNA gene, partial sequence	Oscillospiraceae ...	1855	1855	100%	0.0	99.80%	1455	OP540270.1
Uncultured bacterium clone ncd1060a10c1 16S ribosomal RNA gene, partial sequence	uncultured bacte...	1853	1853	100%	0.0	99.80%	1358	HM344624.1
Uncultured bacterium clone ncd1005h09c1 16S ribosomal RNA gene, partial sequence	uncultured bacte...	1853	1853	100%	0.0	99.80%	1358	HM341026.1
Uncultured bacterium clone ncd998a04c1 16S ribosomal RNA gene, partial sequence	uncultured bacte...	1853	1853	100%	0.0	99.80%	1358	HM332641.1
Uncultured bacterium partial 16S rRNA gene, isolate BF0002B065	uncultured bacte...	1853	1853	100%	0.0	99.80%	1490	AM697115.1
Uncultured bacterium clone nck198e03c1 16S ribosomal RNA gene, partial sequence	uncultured bacte...	1847	1847	100%	0.0	99.70%	1358	KF094825.1
Uncultured bacterium clone ncd1057f07c1 16S ribosomal RNA gene, partial sequence	uncultured bacte...	1847	1847	100%	0.0	99.70%	1358	HM340507.1
Uncultured bacterium clone rRNA351 16S ribosomal RNA gene, partial sequence	uncultured bacte...	1847	1847	100%	0.0	99.70%	1510	AY959124.1
Uncultured bacterium clone ncd1008a01c1 16S ribosomal RNA gene, partial sequence	uncultured bacte...	1845	1845	100%	0.0	99.70%	1357	HM341161.1

Supplemental file 1.3: BLAST alignment analysis of BVAB-2

Oscillospiraceae bacterium strain CHIC02 1186E3-8 16S ribosomal RNA gene, partial sequence

Sequence ID: OP540273.1 Length: 1450 Number of Matches: 1

Range 1: 358 to 1366 GenBank Graphics ▼ Next Match ▲ Previous Match

Score	Expect	Identities	Gaps	Strand
1858 bits(1006)	0.0	1008/1009(99%)	0/1009(0%)	Plus/Plus

```
Query  1     TGGGGAATATTGGGCAATGGGCGAAAGCCTGACCCAGCAACGCCGCGTGAGTGATGAAGG  60
             ||||||||||||||||||||||||||||||||||||||||||||||||||||||||||||
Sbjct  358   TGGGGAATATTGGGCAATGGGCGAAAGCCTGACCCAGCAACGCCGCGTGAGTGATGAAGG  417

Query  61    CCTTCGGGTTGTAAAACTCTTTGGACAGGGACGAAGAAAGTGACGGTACCTGTAGAACAA  120
             ||||||||||||||||||||||||||||||||||||||||||||||||||||||||||||
Sbjct  418   CCTTCGGGTTGTAAAACTCTTTGGACAGGGACGAAGAAAGTGACGGTACCTGTAGAACAA  477

Query  121   GCCACGGCTAACTACGTGCCAGCAGCCGCGGTAATACGTAGGTGGCGAGCGTTATCCGGA  180
             ||||||||||||||||||||||||||||||||||||||||||||||||||||||||||||
Sbjct  478   GCCACGGCTAACTACGTGCCAGCAGCCGCGGTAATACGTAGGTGGCGAGCGTTATCCGGA  537

Query  181   TTTACTGGGCGTAAAGGGCGTGTAGGCGGCTAGATAAGTGTGATGTTTAAATCCAAGGCT  240
             ||||||||||||||||||||||||||||||||||||||||||||||||||||||||||||
Sbjct  538   TTTACTGGGCGTAAAGGGCGTGTAGGCGGCTAGATAAGTGTGATGTTTAAATCCAAGGCT  597

Query  241   TAACCTTGGGGTTCATTACAAACTGTTTAGCTTGAGTGCTGGAGAGGATAGTGGAATTCC  300
             ||||||||||||||||||||||||||||||||||||||||||||||||||||||||||||
Sbjct  598   TAACCTTGGGGTTCATTACAAACTGTTTAGCTTGAGTGCTGGAGAGGATAGTGGAATTCC  657

Query  301   TAGTGTAGCGGTAAAATGCGTAGATATTAGGAGGAACACCGGTGGCGAAGGCGGCTATCT  360
             ||||||||||||||||||||||||||||||||||||||||||||||||||||||||||||
Sbjct  658   TAGTGTAGCGGTAAAATGCGTAGATATTAGGAGGAACACCGGTGGCGAAGGCGGCTATCT  717

Query  361   GGACAGTAACTGACGCTGAGGCGCGAAAGCGTGGGGAGCAAACAGGATTAGATACCCTGG  420
             ||||||||||||||||||||||||||||||||||||||||||||||||||||||||||||
Sbjct  718   GGACAGTAACTGACGCTGAGGCGCGAAAGCGTGGGGAGCAAACAGGATTAGATACCCTGG  777

Query  421   TAGTCCACGCCGTAAACGATGAATACTAGCTGTAGGAGGTATCGACCCCTTCTGTGGCGC  480
             ||||||||||||||||||||||||||||||||||||||||||||||||||||||||||||
Sbjct  778   TAGTCCACGCCGTAAACGATGAATACTAGCTGTAGGAGGTATCGACCCCTTCTGTGGCGC  837

Query  481   AGTTAACACAATAAGTATTCCGCCTGGGGAGTACGGCCGCAAGGTTAAAACTCAAAGGAA  540
             ||||||||||||||||||||||||||||||||||||||||||||||||||||||||||||
Sbjct  838   AGTTAACACAATAAGTATTCCGCCTGGGGAGTACGGCCGCAAGGTTAAAACTCAAAGGAA  897

Query  541   TTGACGGGGACCCGCACAAGCAGTGGATTATGTGGTTTAATTCGAAGCAACGCGAAGAAC  600
             ||||||||||||||||||||||||||||||||||||||||||||||||||||||||||||
Sbjct  898   TTGACGGGGACCCGCACAAGCAGTGGATTATGTGGTTTAATTCGAAGCAACGCGAAGAAC  957

Query  601   CTTACCAGGACTTGACATCCTCTGACGATTCAGGAGACTGAATTTTCTCTTCGGAGACAG  660
             ||||||||||||||||||||||||||||||||||||||||||||||||||||||||||||
Sbjct  958   CTTACCAGGACTTGACATCCTCTGACGATTCAGGAGACTGAATTTTCTCTTCGGAGACAG  1017

Query  661   AGAGACAGGTGGTGCATGGTTGTCGTCAGCTCGTGTCGTGAGATGTTGGGTTAAGTCCCG  720
             ||||||||||||||||||||||||||||||||||||||||||||||||||||||||||||
```

Supplemental file 1.4: BLAST alignment analysis of BVAB-2.

Sequences producing significant alignments Download Select columns Show 100

select all *100 sequences selected* GenBank Graphics Distance tree of results MSA Viewer

Description	Scientific Name	Max Score	Total Score	Query Cover	E value	Per. Ident	Acc. Len	Accession
Clostridiales genomosp. BVAB3 16S ribosomal RNA gene, partial sequence	Mageeibacillus indolicus	1866	1866	100%	0.0	100.00%	1382	KC311734.1
Bacterium BVAB3-Strain 4 16S ribosomal RNA gene, partial sequence	bacterium BVAB3-Strain 4	1866	1866	100%	0.0	100.00%	1480	GQ900634.1
Mageeibacillus indolicus strain 0009-5 16S ribosomal RNA, partial sequence	Mageeibacillus indolicus	1866	1866	100%	0.0	100.00%	1480	NR_146698.1
Uncultured bacterium clone 1123b-58-f 16S ribosomal RNA gene, partial sequence	uncultured bacterium	1866	1866	100%	0.0	100.00%	1503	GQ900629.1
Mageeibacillus indolicus UPII9-5 chromosome, complete genome	Mageeibacillus indolicus ...	1866	3732	100%	0.0	100.00%	1809746	CP001850.2
Uncultured bacterium clone nbw1012c09c1 16S ribosomal RNA gene, partial sequence	uncultured bacterium	1866	1866	100%	0.0	100.00%	1349	GQ043025.1
Uncultured bacterium clone 123f 17 16S ribosomal RNA gene, partial sequence	uncultured bacterium	1866	1866	100%	0.0	100.00%	1010	AY724741.1
Mageeibacillus indolicus strain 0019-D 16S ribosomal RNA gene, partial sequence	Mageeibacillus indolicus	1862	1862	100%	0.0	99.90%	1480	GQ900633.1
Mageeibacillus indolicus strain 0004-9 16S ribosomal RNA gene, partial sequence	Mageeibacillus indolicus	1862	1862	100%	0.0	99.90%	1480	GQ900631.1
Uncultured bacterium clone 8.001-2 16S ribosomal RNA gene, partial sequence	uncultured bacterium	1860	1860	100%	0.0	99.90%	1297	JX871279.1
Uncultured bacterium clone nbu407b07c1 16S ribosomal RNA gene, partial sequence	uncultured bacterium	1855	1855	100%	0.0	99.80%	1349	GQ038494.1
Uncultured bacterium clone FX93B4-11 16S ribosomal RNA gene, partial sequence	uncultured bacterium	1820	1820	98%	0.0	99.80%	1323	AY995273.1
Uncultured bacterium clone WDoral5F08 16S ribosomal RNA gene, partial sequence	uncultured bacterium	1666	1666	100%	0.0	96.44%	1350	KC259695.1
Uncultured bacterium clone 112Q-85 16S ribosomal RNA gene, partial sequence	uncultured bacterium	1663	1663	89%	0.0	100.00%	900	GQ900636.1
Uncultured bacterium clone 2BV-25 16S ribosomal RNA gene, partial sequence	uncultured bacterium	1663	1663	89%	0.0	100.00%	900	EU188978.1
Uncultured bacterium clone WD9oral2D09 16S ribosomal RNA gene, partial sequence	uncultured bacterium	1661	1661	100%	0.0	96.34%	1350	KC260938.1
Uncultured bacterium clone WD9oral2A05 16S ribosomal RNA gene, partial sequence	uncultured bacterium	1661	1661	100%	0.0	96.34%	1350	KC260907.1
Uncultured bacterium clone WD2oralAB03 16S ribosomal RNA gene, partial sequence	uncultured bacterium	1661	1661	100%	0.0	96.34%	1350	KC260805.1
Uncultured bacterium clone WD9oralEH06 16S ribosomal RNA gene, partial sequence	uncultured bacterium	1661	1661	100%	0.0	96.34%	1350	KC260441.1
Uncultured bacterium clone WD1oralAH04 16S ribosomal RNA gene, partial sequence	uncultured bacterium	1661	1661	100%	0.0	96.34%	1351	KC260194.1
Uncultured bacterium clone WDoral5G03 16S ribosomal RNA gene, partial sequence	uncultured bacterium	1661	1661	100%	0.0	96.34%	1351	KC259699.1
Uncultured bacterium clone WDoral3G06 16S ribosomal RNA gene, partial sequence	uncultured bacterium	1661	1661	100%	0.0	96.34%	1350	KC259558.1
Uncultured bacterium clone WD9oral2A07 16S ribosomal RNA gene, partial sequence	uncultured bacterium	1655	1655	100%	0.0	96.24%	1350	KC260908.1
Uncultured bacterium clone WD2oralBG02 16S ribosomal RNA gene, partial sequence	uncultured bacterium	1655	1655	100%	0.0	96.24%	1350	KC260241.1
Uncultured bacterium clone WD1oralAD01 16S ribosomal RNA gene, partial sequence	uncultured bacterium	1655	1655	100%	0.0	96.24%	1350	KC260162.1

Supplemental file 1.5: BLAST alignment analysis of BVAB-3

Download GenBank Graphics Sort by: E value

Mageeibacillus indolicus UPII9-5 chromosome, complete genome

Sequence ID: CP001850.2 Length: 1809746 Number of Matches: 2

Range 1: 255893 to 256902 GenBank Graphics ▼ Next Match ▲ Previous Match

Score	Expect	Identities	Gaps	Strand
1866 bits(1010)	0.0	1010/1010(100%)	0/1010(0%)	Plus/Plus

```
Query  1       TGGGGAATATTGGGCAATGGGCGAAAGCCTGACCCAGCAACGCCGCGTGAAGTATGAAGG  60
               ||||||||||||||||||||||||||||||||||||||||||||||||||||||||||||
Sbjct  255893  TGGGGAATATTGGGCAATGGGCGAAAGCCTGACCCAGCAACGCCGCGTGAAGTATGAAGG  255952

Query  61      CCTTCGGGTTGTAAACTTCTTTGATCAGGGAAGAAACAAATGACGGTACCTGAAAAACAA  120
               ||||||||||||||||||||||||||||||||||||||||||||||||||||||||||||
Sbjct  255953  CCTTCGGGTTGTAAACTTCTTTGATCAGGGAAGAAACAAATGACGGTACCTGAAAAACAA  256012

Query  121     GCCACGGCTAACTACGTGCCAGCAGCCGCGGTAATACGTAGGTGGCGAGCGTTATCCGGA  180
               ||||||||||||||||||||||||||||||||||||||||||||||||||||||||||||
Sbjct  256013  GCCACGGCTAACTACGTGCCAGCAGCCGCGGTAATACGTAGGTGGCGAGCGTTATCCGGA  256072

Query  181     TTTACTGGGTGTAAAGGGCGTGCAGGCGGGCTGATAAGTCAGATGTGAAATCCCCGAGCT  240
               ||||||||||||||||||||||||||||||||||||||||||||||||||||||||||||
Sbjct  256073  TTTACTGGGTGTAAAGGGCGTGCAGGCGGGCTGATAAGTCAGATGTGAAATCCCCGAGCT  256132

Query  241     TAACTCGGGAACTGCATCTGATACTGTTGGTCTTGAGTGCTGGAGAGGATAGTGGAATTC  300
               ||||||||||||||||||||||||||||||||||||||||||||||||||||||||||||
Sbjct  256133  TAACTCGGGAACTGCATCTGATACTGTTGGTCTTGAGTGCTGGAGAGGATAGTGGAATTC  256192

Query  301     CTAGTGTAGCGGTAAAATGCGCAGATATTAGGAGGAACACCAGTGGCGAAGGCGGCTATC  360
               ||||||||||||||||||||||||||||||||||||||||||||||||||||||||||||
Sbjct  256193  CTAGTGTAGCGGTAAAATGCGCAGATATTAGGAGGAACACCAGTGGCGAAGGCGGCTATC  256252

Query  361     TGGACAGTAACTGACGCTGAGGCGCGAAAGCGTGGGTAGCAAACAGGATTAGATACCCTG  420
               ||||||||||||||||||||||||||||||||||||||||||||||||||||||||||||
Sbjct  256253  TGGACAGTAACTGACGCTGAGGCGCGAAAGCGTGGGTAGCAAACAGGATTAGATACCCTG  256312

Query  421     GTAGTCCACGCCGTAAACGATGATTACTAGGTGTAGGAGGTATCGACCCCTTCTGTGCCG  480
               ||||||||||||||||||||||||||||||||||||||||||||||||||||||||||||
Sbjct  256313  GTAGTCCACGCCGTAAACGATGATTACTAGGTGTAGGAGGTATCGACCCCTTCTGTGCCG  256372

Query  481     GAGTTAACACAATAAGTAATCCACCTGGGGAGTACGGCCGCAAGGTTGAAACTCAAAGGA  540
               ||||||||||||||||||||||||||||||||||||||||||||||||||||||||||||
Sbjct  256373  GAGTTAACACAATAAGTAATCCACCTGGGGAGTACGGCCGCAAGGTTGAAACTCAAAGGA  256432

Query  541     ATTGACGGGGGCCCGCACAAGCAGTGGAGTATGTGGTTTAATTCGACGCAACGCGAAGAA  600
               ||||||||||||||||||||||||||||||||||||||||||||||||||||||||||||
Sbjct  256433  ATTGACGGGGGCCCGCACAAGCAGTGGAGTATGTGGTTTAATTCGACGCAACGCGAAGAA  256492

Query  601     CCTTACCAGGGTTTGACATCCCTTGAACGATGTAGAGATACATAATTCCCTTCGGGGACA  660
               ||||||||||||||||||||||||||||||||||||||||||||||||||||||||||||
Sbjct  256493  CCTTACCAGGGTTTGACATCCCTTGAACGATGTAGAGATACATAATTCCCTTCGGGGACA  256552

Query  661     AGGAGACAGGTGGTGCATGGTTGTCGTCAGCTCGTGTCGTGAGATGTTGGGTTAAGTCCC  720
```

Supplemental file 1.6: BLAST alignment analysis of BVAB-3
