# Supplementary figures and images for: Species-Specific Analysis of Bacterial Vaginosis-Associated Bacteria
Source: Microbiol Spectr. 2023 Jun 22;11(4):e04676-22. doi: 10.1128/spectrum.04676-22 (PMC10434177; doi:10.1128/spectrum.04676-22)

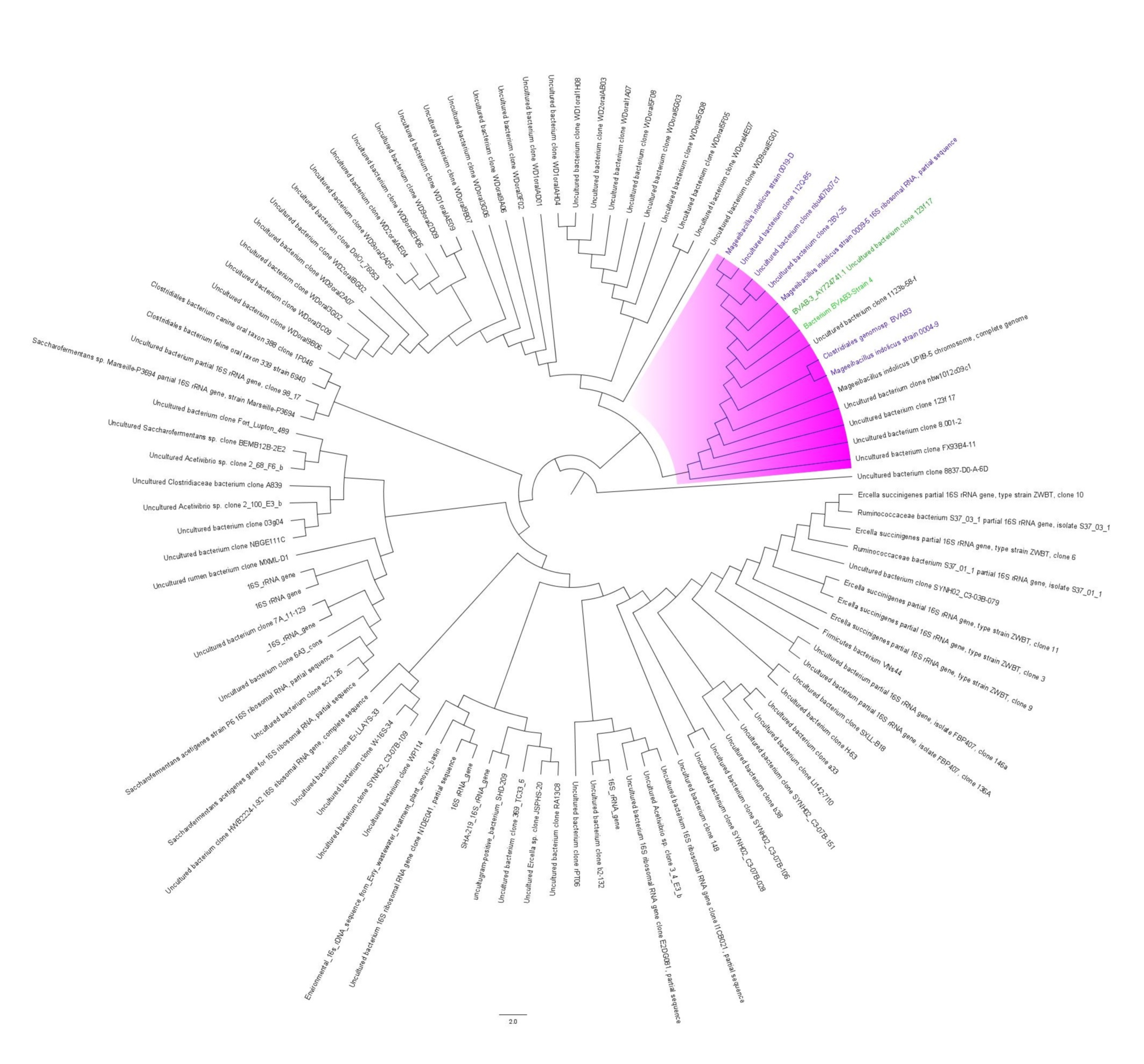

Supplement: Supplemental file 2 — Supplemental material. Download spectrum.04676-22-s0002.jpg, JPG file, 2.0 MB [file spectrum.04676-22-s0002.jpg]

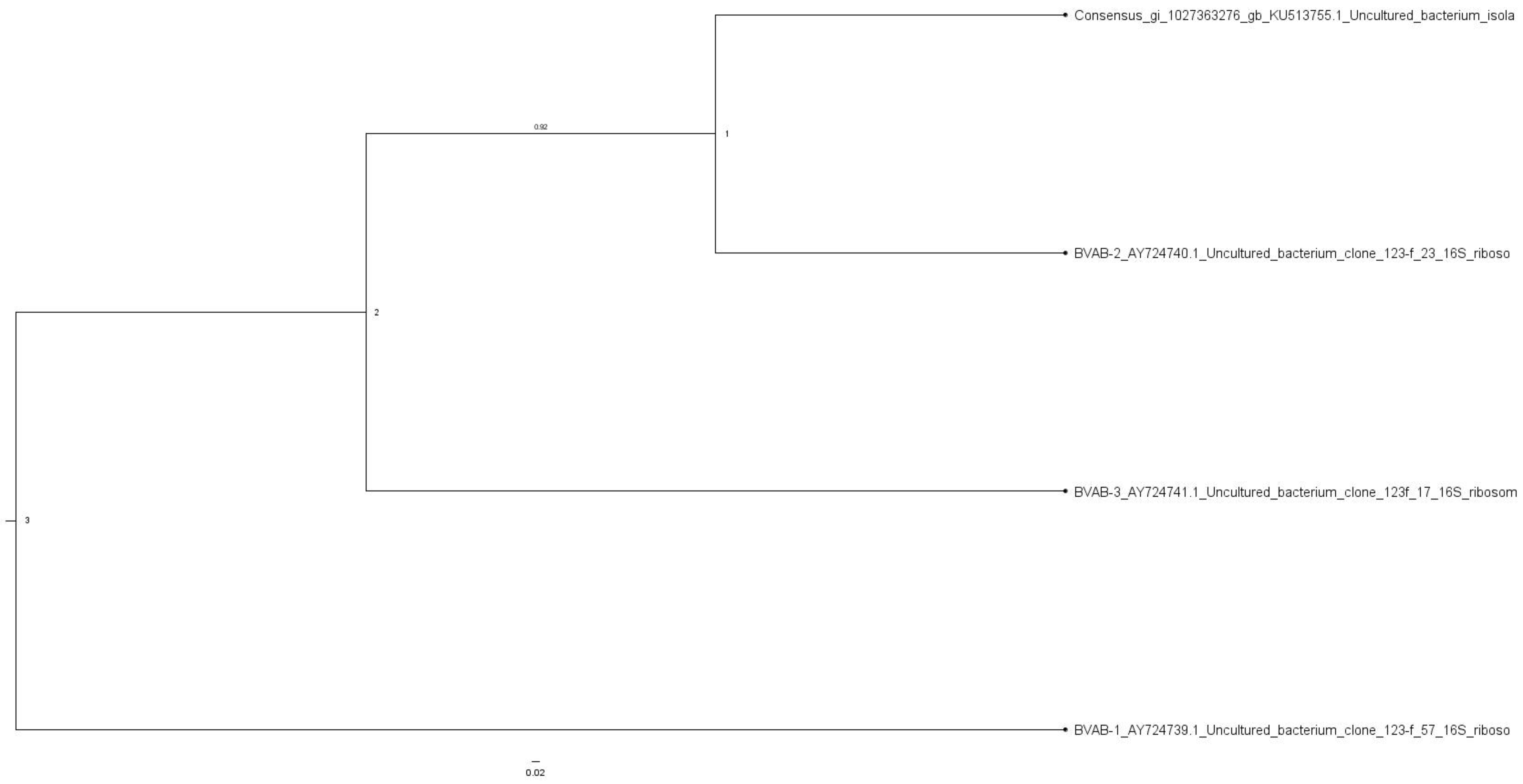

Supplement: Supplemental file 3 — Supplemental material. Download spectrum.04676-22-s0003.jpg, JPG file, 0.4 MB [file spectrum.04676-22-s0003.jpg]
