# Supplementary material for: Species-Specific Analysis of Bacterial Vaginosis-Associated Bacteria
Source: Microbiol Spectr. 2023 Jun 22;11(4):e04676-22. doi: 10.1128/spectrum.04676-22 (PMC10434177; doi:10.1128/spectrum.04676-22)
Supplement: Supplemental file 4 — Supplemental material. Download spectrum.04676-22-s0004.docx, DOCX file, 0.01 MB [file spectrum.04676-22-s0004.docx]

**Figure S1. Phylogenetic tree of BVAB-3 and closely related 16s rRNA/genomes.** BVAB-3 is colored in green while other evolutionary closer species or 16S rRNA that are closely related to BVAB-3, i.e., *Mageeibacillus indolicus* strains*,* are colored in violet. The clade of BVAB-3 and other closely related species is highlighted in violet. The close clustering of BVAB-3_AY

**Figure S2. Phyologenetic tree showing the relationship of BVAB-1, -2, and -3.**

**Supplemental file 1**. BLAST sequence homology and length coverage analyses of BVAB-1, BVAB-2, and BVAB-3.
